# High Neural Efficiency in Unconscious Perceptual Processing among Table Tennis Athletes: An Event-Related Potential Study

**DOI:** 10.3390/brainsci14080756

**Published:** 2024-07-27

**Authors:** Jilong Shi, Haojie Huang, Fatima A. Nasrallah, Anmin Li

**Affiliations:** 1 School of Psychology, Shanghai University of Sport, Shanghai 200438, China; shijilong096@163.com; 2 Department of Physical Education, Xiamen University, Xiamen 361005, China; huanghaojie24@xmu.edu.cn; 3 Queensland Brain Institute, The University of Queensland, Saint Lucia, QLD 4067, Australia; f.nasrallah@uq.edu.au

**Keywords:** table tennis athletes, neural efficiency, unconscious perceptual processing, masked priming paradigm, event-related potential

## Abstract

Background: Neural efficiency refers to the brain’s ability to function with reduced resource expenditure while maintaining high performance levels. Previous research has demonstrated that table tennis athletes have greater neural efficiency at the conscious level. However, it is unknown whether they exhibit greater neural efficiency at the unconscious level. Therefore, this study aims to investigate unconscious perceptual processing and neural efficiency in elite table tennis athletes through tasks involving the judgment of spin serves. Methods: Fifty healthy, right-handed individuals participated in this study, including 25 elite table tennis athletes and 25 control participants without professional training experience. To evaluate the unconscious perceptual characteristics of both groups, we used a combination of masked priming paradigm and event-related potential techniques. Results: The behavioral results reveal that, compared to the control group, the table tennis athletes displayed reduced reaction times (*p* < 0.001) and increased priming effects (*p* < 0.001) under unconscious conditions. The electrophysiological findings indicated that both groups elicited N1, N2, and P2 components. Notably, compared to the control group, the table tennis athletes exhibited significantly lower amplitude responses at the occipital lobe electrodes PO3, POz, PO4, O1, Oz, and O2 (*p* < 0.001). Conclusions: These results further support the neural efficiency hypothesis, indicating that prolonged professional training enhances athletes’ capacities for specialized unconscious cognitive processing.

## 1. Introduction

Neural efficiency refers to the brain’s capacity to function with minimal resource expenditure while maintaining high levels of performance [1]. Originating from the neural efficiency hypothesis proposed by Haier et al. (1988), this concept has been observed in various domains of expertise [2]. For example, athletes often demonstrate more efficient neural activity compared to non-athletes, implying that extensive practice allows experts to perform tasks with less neural activation, optimizing brain resource use [3,4]. This reduced activation leads to quicker and more accurate responses, which are advantageous in competitive environments like sports. A study has shown that compared to non-athletes, table tennis athletes (TTAs) not only exhibit greater neural efficiency in visuospatial tasks but also demonstrate this through reduced functional magnetic resonance imaging activation [5]. Additionally, electroencephalogram (EEG) studies on TTAs have found reduced task-related negative potential amplitude for incongruent trials in the frontal cortex, reduced late sustained potential amplitudes in the parietal cortex, and reduced α event-related desynchronization in the mid-parietal region compared to non-athletes [6]. These findings support the neural efficiency hypothesis, suggesting that experienced athletes perform cognitive tasks with less neural activation due to highly efficient neural networks developed through extensive training [7]. This efficiency boosts reaction speed and accuracy, reducing cognitive load and enabling athletes to maintain peak performance under pressure [8].

However, some research contradicts this hypothesis. For instance, TTAs have been found to exhibit higher N1, N2, and P3 amplitudes in the frontal and central regions [6,9], as well as stronger functional connectivity between the right occipital–temporal and right frontal–temporal areas [10]. Given that these studies utilized different experimental tasks, the inconsistency in their findings may indicate that the complexity and characteristics of these tasks influence the outcomes [11].

It is important to note that the studies mentioned above have all focused on the conscious awareness level in TTAs. However, in actual matches, the unconscious perceptual abilities of TTAs are crucial [12,13]. This capability enables athletes to process and respond to external visual stimuli rapidly and accurately in a fast-paced, dynamic competitive environment. Unconscious perception involves the brain’s automatic processing of information without conscious effort, allowing athletes to concentrate on strategy and technical execution [14]. Typically, unconscious perception is studied using the masked priming paradigm [14]. In this context, “masked” refers to the technique of presenting a visual stimulus with a preceding or following interfering stimulus, thus making the priming stimulus hidden. “Priming” describes how an initial cue stimulus influences the processing of a subsequent target stimulus [14]. Previous studies suggest that the sandwich masked priming paradigm, which incorporates both forward (presenting the masked stimulus before the priming stimulus) and backward masking (presenting the masked stimulus after the priming stimulus), exhibits superior efficacy in masking effects compared to either the forward or backward masked priming paradigm alone [15,16].

Behavioral studies using the sandwich masked priming paradigm have shown that TTAs possess significant perceptual advantages over non-athletes, even with increased perceptual loads [7,13,17,18]. These findings underscore the unconscious perceptual benefits that TTAs possess. In event-related potential (ERP) studies, research demonstrates that TTAs exhibit a perceptual advantage in unconscious response inhibition tasks (go/no go) and unconscious flanker tasks (flanker and masked prime) [7,17]. Compared to non-athletes, TTAs manifest significantly larger amplitudes in the N1, N2, P1, and P3 components [7,17]. These findings contradict the neural efficiency hypothesis. We speculate that the discrepancies may arise from the nature of the stimulus materials employed; both studies utilized arrows or simple geometric shapes unrelated to sport-specific scenarios as stimuli.

Despite some understanding of the perceptual processing traits of TTAs, there are currently no studies investigating the unconscious perceptual capabilities of TTAs using stimuli relevant to real sporting contexts, especially in tasks involving table tennis serve rotation. In table tennis matches, rapid changes in the competitive environment make it challenging for athletes to consciously and accurately judge the spin direction of the ball, often leading to reliance on unconscious judgments [19]. Additionally, previous research indicates that TTAs typically determine spin direction by observing the contact point between the paddle and the ball [20,21]. Given this, our study aims to address two primary questions: first, do TTAs possess unconscious perceptual advantages in spin serve judgment tasks? Second, do TTAs demonstrate high neural efficiency at the unconscious level? Accordingly, we propose the following hypotheses: first, TTAs possess significant unconscious perceptual advantages in judging the spin direction of serves compared to non-athletes. Second, TTAs exhibit higher neural efficiency at the unconscious level during spin serve judgment tasks, as evidenced by reduced neural activation in relevant brain areas compared to non-athletes. To investigate these hypotheses, we used stimuli that closely approximate real table tennis match conditions, moving beyond the arrows or simple geometric shapes unrelated to sport-specific scenarios utilized in previous research [7,17]. By exploring the unconscious perceptual capabilities and neural efficiency of TTAs, this study seeks to bridge current research gaps and deepen our understanding of the perceptual processing characteristics and neural efficiency of TTAs in unconscious states.

## 2. Materials and Methods

### 2.1. Participants

This study was conducted at the Center for Exercise and Brain Science of Shanghai University of Sport. Participants were recruited through online advertisements at Shanghai Sports University and participated anonymously on a voluntary basis. All the recruited participants agreed to partake in the experiments without refusal. The inclusion criteria required the participants to have normal or corrected-to-normal vision and no psychological or neurological disorders. For the TTA group, eligibility required intensive training in table tennis over the preceding three months and the achievement of at least the first level according to the Chinese national standard. The non-athlete group consisted of individuals with no prior training in table tennis or any other racket sports. Additionally, the participants confirmed that they had not participated in similar experiments before this study. They reported no previous neurological, psychiatric, cardiovascular, or musculoskeletal diseases, nor had they taken centrally acting drugs before the experiment.

G*Power3 (Heinrich-Heine-Universität Düsseldorf, Düsseldorf, North Rhine-Westphalia, Germany, 2007) as used to determine the required sample size [22]. With an effect size of 0.25, a confidence level of 0.95, and a statistical power of 0.95, assuming a correlation coefficient of 0.5 for repeated measures, a 2 × 3 repeated measures ANOVA indicated that at least 44 participants were needed, with 22 per group. To further enhance the statistical robustness, we ultimately recruited 50 participants. The first group consisted of 25 elite TTAs (12 females), averaging 19.84 ± 1.98 years in age and 9.12 ± 3.77 years in training experience, who were selected due to their extensive table tennis practice. The comparison group (CG) consisted of 25 non-athletes (12 females), with an average age of 19.76 ± 1.81 years, who were selected for their lack of experience in table tennis or similar racket sports. This study was approved by the Shanghai University of Sport’s ethics committee and conducted in accordance with the latest Declaration of Helsinki.

### 2.2. Stimulus Material

In real-match scenarios, athletes assess the ball’s spin by noting where the racket meets the ball; this is a crucial ability for gaining an edge in elite competitions. By concentrating on variations in the racket–ball contact point, athletes can accurately identify the spin’s direction and force. Different contact points indicate different spins. For instance, bottom contact might imply topspin, while top contact could suggest backspin. By noting these minor variations, athletes can react swiftly and precisely.

**Figure 1:**
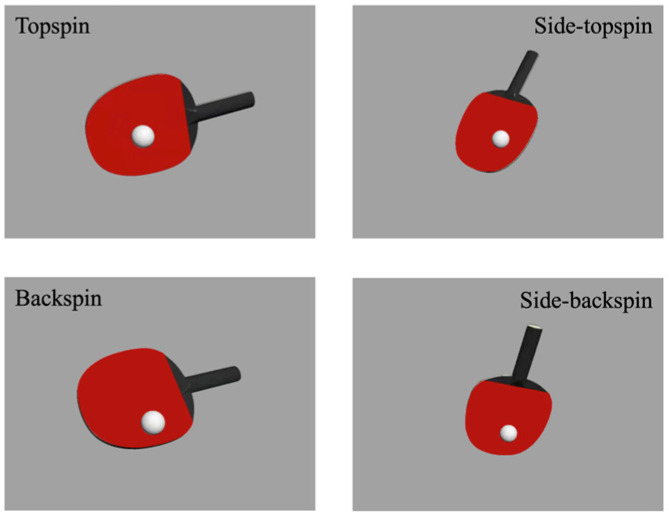
Priming stimuli and target stimuli materials, including four types of spinning balls in table tennis: topspin, backspin, side-topspin, and side-backspin.

Using kinematics and sports biomechanics principles, and analyzing self-recorded table tennis serve videos, we used 3ds Max 2012 software (https://www.autodesk.com/products/3ds-max/free-trial: 1 December 2022) for simulations to generate detailed models of table tennis serves, focusing on the ball–racket contact. The images were modeled with precision using real-life parameters to ensure the simulations’ authenticity. Throughout the modeling process, the specific parameters of the actual objects were meticulously adhered to, with special attention paid to accurately modeling the table tennis ball and racket. Using these parameters, four serve spin images were produced as both priming and target stimuli, covering topspin, backspin, side-topspin, and side-backspin, as depicted in Figure 1. Additionally, to achieve effective masking, masking images were created with multiple black and red lines.

### 2.3. Task

The participants were seated 60 cm away from a 24-inch LCD monitor (resolution: 1920 × 1080 pixels; refresh rate: 60 Hz) and instructed to press the “F” and “J” keys on a keyboard to respond during the experiment. E-prime software (Version 3, Psychology Software Tools, Inc., Sharpsburg, PA, USA) was used for stimulus presentation, behavioral response recording, and EEG synchronization. A practice task was included to ensure that the participants were familiar with the tasks before starting the experimental phase.

**Figure 2:**
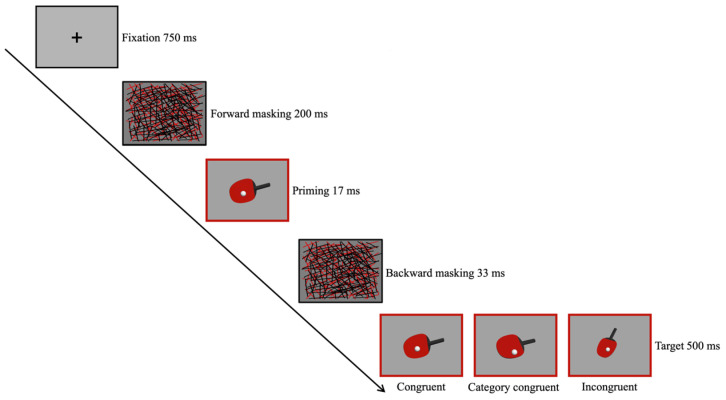
Schematic illustration of the masked priming paradigm.

The design employed a masked priming paradigm to explore unconscious perceptual processing. Each trial began with a black “+” symbol displayed for 750 ms at the center of a gray screen, followed by a forward mask of black and red lines for 200 ms. A priming stimulus was then presented for 17 ms, followed by a backward mask of black and red lines and, finally, the target stimulus. The participants judged the direction based on the target stimulus, pressing “F” for topspin or backspin, and “J” for side-topspin or side-backspin. The inter-stimulus interval (ISI) between a response and the next trial varied randomly from 1000 ms to 1200 ms, as illustrated in Figure 2.

Four types of serve spins were used for both priming and target stimuli, resulting in sixteen combinations. To ensure even representation of each combination, the number of trials was set as a multiple of 16. The experiment was divided into 4 blocks of 48 trials each, totaling 192 trials. If the priming and target stimuli were identical, the trial was considered a congruent condition. If the priming and target stimuli belonged to the same category (e.g., topspin priming with backspin target or side-topspin priming with side-backspin target), it was marked as a category congruent condition. All the other scenarios were labeled as incongruent conditions. At the end of each block, the participants had a minimum rest period of 1 min.

Upon completion, the participants were asked about their conscious perception of the prime stimulus; specifically, they were asked whether they noticed any visual stimulus before the mask. Additionally, to evaluate prime visibility, an objective measurement of prime identification was conducted after completing the unconscious priming task [23]. The participants were informed about the prime’s existence. This task comprised 64 trials, following the same procedure as the unconscious priming task. The masked prime elicited either the same or a different response compared to the subsequent target. The participants were required to identify the spin of the prime’s serve without any time constraints. No feedback was provided during this task.

### 2.4. EEG Recording and Analysis

The EEG signals were recorded using Brain Vision Recorder, version 2.0 (Brain Products GmbH, Gilching, Germany), with a 1000 Hz sampling rate from 64 electrodes positioned on the scalp according to the international 10-10 system. The EEG record was referenced online against the FCz site and was grounded at the AFz site. An electrode was placed on each mastoid (left and right) for offline re-referencing. The vertical electrooculogram was recorded just below the left eye. The horizontal electrooculogram was recorded at the outer canthus of the right eye. Electrode impedances were kept below 5 kΩ for the duration of the experiment.

After recording, the EEG data were processed and analyzed using EEGLAB14 (https://sccn.ucsd.edu/eeglab/index.php: 15 December 2023), an open-source MATLAB toolbox designed for neurophysiological data analysis. The EEG data were high-pass filtered with 0.1 Hz for ERP analysis and low-pass filtered with 30 Hz (Butterworth filter, slope: 24 dB/oct). Additionally, notch filters at 48 Hz and 52 Hz were applied to eliminate noise. Ocular artifacts and any other residual artifacts were isolated through independent component analysis and visual inspection. For ERP analysis, continuous EEG data were segmented from 200 ms before to 1200 ms after the onset of the follow-up stimulus. All the trials, except those with large artifacts (amplitudes exceeding ± 100μV), were included in the analyses. Over 97% of the trials were retained for each condition. The statistical results of the number of remaining trials showed no significant difference between the two groups (t_(24)_ = −0.004, *p* = 0.996).

The ERP data were baseline-corrected using data obtained from −450 ms to −250 ms. Recent research suggests that the N1, N2, and P2 components play roles in unconscious processing [19,24,25]. Consequently, we analyzed mean amplitudes for the N1, N2, and P2 components within the 100–120 ms, 150–170 ms, and 190–210 ms time windows.

### 2.5. Statistics

The statistical analyses were performed using SPSS 27.0 (IBM Corp. Released 2020. IBM SPSS Statistics for Windows, Version 27.0. IBM Corp: Armonk, NY, USA). Only correct trials with reaction times (RTs) ranging from 100 to 1000 ms post-target onset were included in the analysis. Mixed-effects ANOVAs, incorporating both within-subject and between-subject factors, were conducted to analyze behavioral (hit RTs and error rates) and neurophysiological (prime target-evoked N1, P2, and N2) data. The models incorporated the between-subject factor ‘group’ (TTAs and control) and the within-subject factors ‘prime congruency’ (congruent, category congruent, and incongruent). The degrees of freedom were adjusted using the Greenhouse–Geisser correction, and the post hoc tests underwent Bonferroni correction as needed. For all descriptive statistics, the standard error of the mean (SEM) was reported as a measure of variability. Additionally, partial eta-squared ($\eta_{p}^{2}$) was computed to determine the effect sizes for both the main and interaction effects [26].

## 3. Results

### 3.1. Visibility Assessment Results

In the visibility assessment test, no one spontaneously reported having seen the prime image. When informed of the nature of the prime image, no one could recall any details of it.

Objective measurements were obtained in the forced-choice task. A one-sample t-test revealed that the TTA group’s average accuracy rate in the forced-choice task was 50.94%, which was not significantly different from chance (t_(24)_ = 1.162, *p* = 0.257). Similarly, the CG’s average accuracy rate was 50.13%, which was also not significantly different from chance (t_(24)_ = 0.114, *p* = 0.910). Furthermore, an independent samples t-test comparing the average accuracy rates between the TTAs and CG found no significant difference (t_(48)_ = 0.598, *p* = 0.553).

### 3.2. Behavioral Results

**Figure 3:**
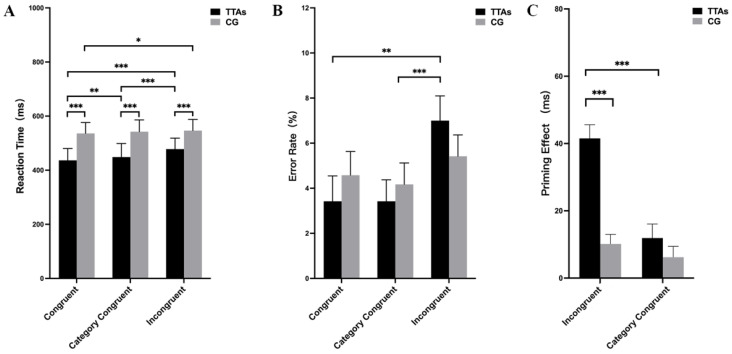
Behavioral results of TTAs and the CG. (**A**) shows RT results; (**B**) shows error rate results; (**C**) shows priming effect; incongruent represents the incongruent priming effect, and category congruent represents the category congruent priming effect. * indicates *p* < 0.05, ** indicates *p* < 0.01, *** indicates *p* < 0.001.

The analysis of the RT data in the mixed-effects ANOVA revealed a statistically significant two-way interaction between group and condition (F_(1, 48)_ = 19.310, *p* < 0.001, $\eta_{p}^{2}$ = 0.287). Additionally, there were main effects of group (F_(1, 48)_ = 53.880, *p* < 0.001, $\eta_{p}^{2}$ = 0.529) and condition (F_(1, 48)_ = 47.441, *p* < 0.001, $\eta_{p}^{2}$ = 0.497). The simple effects analysis of the groups showed that the TTAs responded faster than the CG in the congruent condition (F_(1, 48)_ = 70.430, *p* < 0.001, $\eta_{p}^{2}$ = 0.595), in the category congruent condition (F_(1, 48)_ = 49.631, *p* < 0.001, $\eta_{p}^{2}$ = 0.508), and in the incongruent condition (F_(1, 48)_ = 34.686, *p* < 0.001, $\eta_{p}^{2}$ = 0.419). The simple effects analysis of the condition revealed that the effect of condition was significant in the TTAs (F_(1, 48)_ = 68.950, *p* < 0.001, $\eta_{p}^{2}$ = 0.746) and was also significant in the CG (F_(1, 48)_ = 4.373, *p* < 0.05, $\eta_{p}^{2}$ = 0.157). Multiple comparisons found that in the TTAs, the RTs in the congruent condition were significantly shorter than in the category congruent condition (*p* < 0.01) and the incongruent condition (*p* < 0.001); the RTs in the category congruent condition were significantly shorter than in the incongruent condition. In the CG, the RTs in the congruent condition were significantly shorter than in the incongruent condition (*p* < 0.05) (Figure 3A).

The analysis of the error rate in the mixed-effects ANOVA revealed a two-way interaction between group and condition that was not statistically significant (F_(1, 48)_ = 2.423, *p* = 0.094, $\eta_{p}^{2}$ = 0.048). However, the main effects analysis showed a significant effect of condition (F_(1, 48)_ = 7.911, *p* < 0.01, $\eta_{p}^{2}$ = 0.142) and a non-significant effect of group (F_(1, 48)_ = 0.015, *p* = 0.903, $\eta_{p}^{2}$ = 0.000). Multiple comparisons found that in the TTAs, the error rates in the incongruent condition were significantly higher than in the congruent condition (*p* < 0.01) and the category congruent condition (*p* < 0.001) (Figure 3B).

The priming effect in the masked priming paradigm can, to some extent, reflect perceptual processing ability [16]. The priming effect is derived by subtracting the RT under the congruent condition or category congruent condition from the RT under the incongruent condition. Due to the increased difficulty of the experimental task in this study, a category congruent condition was introduced. Therefore, there are two priming effects in this study: one is the incongruent priming effect, obtained by subtracting the RT under the congruent condition from the RT under the incongruent condition; the other is the category congruent priming effect, obtained by subtracting the RT under the category congruent condition from the RT under the incongruent condition.

The analysis of the priming effect data in the mixed-effects ANOVA revealed a statistically significant two-way interaction between the group and priming effects (F_(1, 48)_ = 19.008, *p* < 0.001, $\eta_{p}^{2}$ = 0.284). Additionally, there were the main effects of the group (F_(1, 48)_ = 70.062, *p* < 0.001, $\eta_{p}^{2}$ = 0.593) and priming effects (F_(1, 48)_ = 32.383, *p* < 0.001, $\eta_{p}^{2}$ = 0.403). The simple effects analysis of the groups indicated that the incongruent priming effect for the TTAs was significantly greater than for the CG (F_(1, 48)_ = 40.041, *p* < 0.001, $\eta_{p}^{2}$ = 0.455). Additionally, the simple effects analysis of the priming effect revealed that, within the TTAs, the incongruent priming effect was significantly greater than the category congruent priming effect (F_(1, 48)_ = 48.000, *p* < 0.001, $\eta_{p}^{2}$ = 0.513) (Figure 3C).

### 3.3. Neurophysiological Data

#### 3.3.1. N1 Component

**Figure 4:**
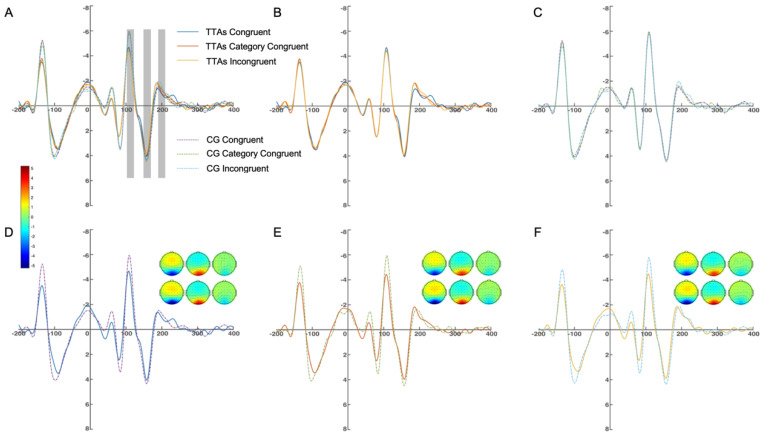
Average amplitude of the O1, Oz, O2 electrodes. (**A**) shows the waveforms for all conditions of both groups, with the shaded areas from left to right corresponding to the N1, P2, and N2 components, respectively. (**B**) shows the waveforms within the TTAs. (**C**) shows the waveforms within the CG. (**D**) shows the comparison of amplitudes between the two groups under congruent conditions. (**E**) shows the comparison of amplitudes between the two groups under category congruent conditions. (**F**) shows the comparison of amplitudes between the two groups under incongruent conditions.

A two-factor repeated measures ANOVA (group and response congruency) was conducted on the mean amplitude of O1, O2, and Oz between 100 and 120 ms. The results indicated that the main effect of group was significant (F_(1, 48)_ = 4.61, *p* < 0.05, $\eta_{p}^{2}$ = 0.20), the main effect of response congruency was significant (F_(1, 48)_ = 36.79, *p* < 0.001, $\eta_{p}^{2}$ = 0.67), and the interaction between group and response congruency was significant (F_(1, 48)_ = 35.34, *p* < 0.001, $\eta_{p}^{2}$ = 0.66). The simple effect analysis of group showed that in the category congruent condition, the simple effect of group was significant (F_(1, 48)_ = 6.18, *p* < 0.05, $\eta_{p}^{2}$ = 0.26); and in the incongruent condition, the simple effect of group was significant (F_(1, 48)_ = 4.54, *p* < 0.05, $\eta_{p}^{2}$ = 0.20). The simple effect analysis of response congruency showed that the simple effect of response congruency was significant in the athlete group (F_(1, 48)_ = 27.73, *p* < 0.001, $\eta_{p}^{2}$ = 0.77) and in the CG (F_(1, 48)_ = 73.82, *p* < 0.001, $\eta_{p}^{2}$ = 0.90). Multiple comparisons found that in the TTAs, the amplitude in the congruent condition was significantly smaller than in the category congruent condition (*p* < 0.001) and the incongruent condition (*p* < 0.001). In the CG, the amplitude in the incongruent condition was significantly smaller than in the congruent condition (*p* < 0.01) and the category congruent condition (*p* < 0.001) (Figure 4).

**Figure 5:**
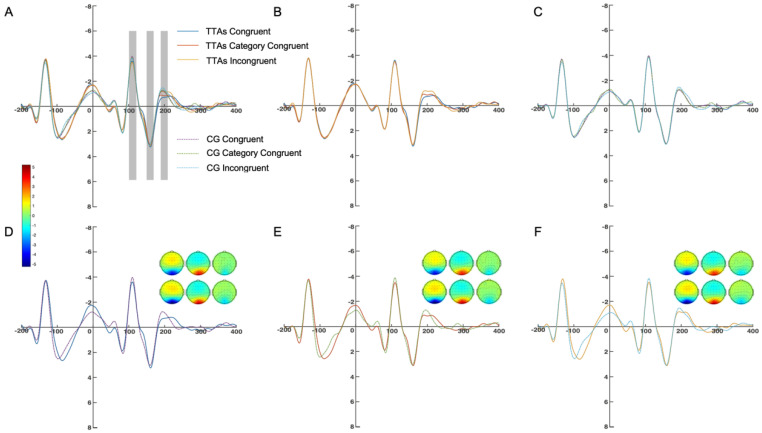
Average amplitude of the PO3, POz, PO4 electrodes. (**A**) shows the waveforms for all conditions of both groups, with the shaded areas from left to right corresponding to the N1, P2, and N2 components, respectively. (**B**) shows the waveforms within the TTAs. (**C**) shows the waveforms within the CG. (**D**) shows the comparison of amplitudes between the two groups under congruent conditions. (**E**) shows the comparison of amplitudes between the two groups under category congruent conditions. (**F**) shows the comparison of amplitudes between the two groups under incongruent conditions.

A two-factor repeated measures ANOVA (group and response congruency) was conducted on the mean amplitude of PO3, PO4, and POz between 100 and 120 ms. The results indicated that the main effect of group was not significant (F_(1, 48)_ = 1.54, *p* = 0.23, $\eta_{p}^{2}$ = 0.08), the main effect of response congruency was significant (F_(1, 48)_ = 24.50, *p* < 0.001, $\eta_{p}^{2}$ = 0.58), and the interaction between group and response congruency was not significant (F_(1, 48)_ = 2.15, *p* = 0.16, $\eta_{p}^{2}$ = 0.10). The simple effect analysis of response congruency showed that the simple effect of response congruency was significant in the athlete group (F_(1, 48)_ = 27.73, *p* < 0.001, $\eta_{p}^{2}$ = 0.77) and in the CG (F_(1, 48)_ = 73.82, *p* < 0.001, $\eta_{p}^{2}$ = 0.90). Multiple comparisons found that in the TTAs, the amplitude in the congruent condition was significantly smaller than in the category congruent condition (*p* < 0.05) and the incongruent condition (*p* < 0.01). In the CG, the amplitude in the incongruent condition was significantly smaller than in the congruent condition (*p* < 0.001) and the category congruent condition (*p* < 0.001) (Figure 5).

#### 3.3.2. P2 Component

A two-factor repeated measures ANOVA (group and response congruency) was conducted on the mean amplitude of O1, O2, and Oz between 150 and 170 ms. The results indicated that the main effect of group was significant (F_(1, 48)_ = 5.57, *p* < 0.05, $\eta_{p}^{2}$ = 0.24), the main effect of response congruency was not significant (F_(1, 48)_ = 0.57, *p* = 0.47, $\eta_{p}^{2}$ = 0.003), and the interaction between group and response congruency was not significant (F_(1, 48)_ = 0.74, *p* = 0.41, $\eta_{p}^{2}$ = 0.04). The simple effect analysis of group showed that in the category congruent condition, the simple effect of group was significant (F_(1, 48)_ = 5.72, *p* < 0.05, $\eta_{p}^{2}$ = 0.24); and in the incongruent condition, the simple effect of group was not significant (F_(1, 48)_ = 6.32, *p* < 0.05, $\eta_{p}^{2}$ = 0.26) (Figure 4).

A two-factor repeated measures ANOVA (group and response congruency) was conducted on the mean amplitude of PO3, PO4, and POz between 150 and 170 ms. The results indicated that the main effect of group was not significant (F_(1, 48)_ = 0.09, *p* = 0.77, $\eta_{p}^{2}$ = 0.01), the main effect of response congruency was not significant (F_(1, 48)_ = 3.05, *p* = 0.09, $\eta_{p}^{2}$ = 0.15), and the interaction between group and response congruency was not significant (F_(1, 48)_ = 0.43, *p* = 0.55, $\eta_{p}^{2}$ = 0.02) (Figure 5).

#### 3.3.3. N2 Component

A two-factor repeated measures ANOVA (group and response congruency) was conducted on the mean amplitude of O1, O2, and Oz between 190 and 210 ms. The results indicated that the main effect of group was significant (F_(1, 48)_ = 7.71, *p* < 0.05, $\eta_{p}^{2}$ = 0.39), the main effect of response congruency was significant (F_(1, 48)_ = 140.22, *p* < 0.001, $\eta_{p}^{2}$ = 0.92), and the interaction between group and response congruency was not significant (F_(1, 48)_ = 1.69, *p* = 0.22, $\eta_{p}^{2}$ = 0.66). The simple effect analysis of group showed that in the congruent condition, the simple effect of group was significant (F_(1, 48)_ = 10.22, *p* < 0.01, $\eta_{p}^{2}$ = 0.46); in the category congruent condition, the simple effect of group was significant (F_(1, 48)_ = 10.88, *p* < 0.01, $\eta_{p}^{2}$ = 0.48). The simple effect analysis of response congruency showed that the simple effect of response congruency was significant in the athlete group (F_(1, 48)_ = 61.37, *p* < 0.001, $\eta_{p}^{2}$ = 0.92) and in the CG (F_(1, 48)_ = 74.57, *p* < 0.001, $\eta_{p}^{2}$ = 0.93). Multiple comparisons found that in the TTAs, the amplitude in the congruent condition was significantly smaller than in the category congruent condition (*p* < 0.001) and the incongruent condition (*p* < 0.001), whereas the amplitude in the category congruent condition was significantly smaller than in the incongruent condition (*p* < 0.001). In the CG, the amplitude in the congruent condition was significantly smaller than in the category congruent condition (*p* < 0.001) and the incongruent condition (*p* < 0.001), whereas the amplitude in the category congruent condition was significantly smaller than in the incongruent condition (*p* < 0.01) (Figure 4).

A two-factor repeated measures ANOVA (group and response congruency) was conducted on the mean amplitude of PO3, PO4, and POz between 190 and 210 ms. The results indicated that the main effect of group was significant (F_(1, 48)_ = 55.15, *p* < 0.001, $\eta_{p}^{2}$ = 0.75), the main effect of response congruency was significant (F_(1, 48)_ = 496.15, *p* < 0.001, $\eta_{p}^{2}$ = 0.97), and the interaction between group and response congruency was significant (F(1, 48) = 66.84, *p* < 0.001, $\eta_{p}^{2}$ = 0.79). The simple effect analysis of group showed that in the congruent condition, the simple effect of group was significant (F_(1, 48)_ = 99.34, *p* < 0.001, $\eta_{p}^{2}$ = 0.85); in the category congruent condition, the simple effect of group was significant (F_(1, 48)_ = 55.26, *p* < 0.001, $\eta_{p}^{2}$ = 0.75); and in the incongruent condition, the simple effect of group was significant (F_(1, 48)_ = 17.27, *p* < 0.01, $\eta_{p}^{2}$ = 0.49). The simple effect analysis of response congruency showed that the simple effect of response congruency was significant in the athlete group (F_(1, 48)_ = 1312.21, *p* < 0.001, $\eta_{p}^{2}$ = 0.99) and in the CG (F_(1, 48)_ = 305.40, *p* < 0.001, $\eta_{p}^{2}$ = 0.97). Multiple comparisons found that in the TTAs, the amplitude in the congruent condition was significantly smaller than in the category congruent condition (*p* < 0.001) and the incongruent condition (*p* < 0.001), whereas the amplitude in the category congruent condition was significantly smaller than in the incongruent condition (*p* < 0.001). In the CG, the amplitude in the congruent condition was significantly smaller than in the category congruent condition (*p* < 0.01) and the incongruent condition (*p* < 0.001), whereas the amplitude in the category congruent condition was significantly smaller than in the incongruent condition (*p* < 0.01) (Figure 5).

## 4. Discussion

This study aimed to address a gap in the existing literature by investigating the unconscious perceptual capabilities of TTAs using stimuli relevant to real sporting contexts, particularly tasks involving table tennis serve rotation. By employing images of table tennis serves with various spins as stimuli and integrating the masked priming paradigm with ERP technology, we explored the unconscious perceptual processing abilities of TTAs. The results addressed the two primary questions posed in the introduction: first, the athletes demonstrated a significant unconscious perceptual advantage in judging serve spins, which was closely linked to their extensive sports experience. Second, the findings revealed that the TTAs exhibited greater neural efficiency compared to non-athletes in the occipital regions during the unconscious processing of spin serve stimuli. These findings are discussed in the subsequent sections.

The behavioral results indicated that under unconscious conditions, the TTAs exhibited a significant expert advantage, with RTs significantly shorter than those of the non-athletes. This may reflect the athletes’ ability to quickly capture critical information about the spin of the serve, allowing them to make accurate decisions more rapidly [27]. This finding highlights the athletes’ superior ability in unconscious perceptual processing, especially in tasks related to their expertise; this is congruent with previous research [17,18]. Unlike previous studies, this study included a neutral condition, the category congruent condition, to further analyze the effect of task difficulty on the unconscious perceptual processing of TTAs. A detailed analysis of response consistency revealed that under unconscious conditions, the athletes’ RTs were shorter in the congruent condition than in the category congruent and incongruent conditions, evoking both an incongruent priming effect and a category congruent priming effect. This suggests that even in the more difficult category congruent condition, subtle changes in the contact point between the paddle and the ball could elicit a priming effect in TTAs [19,21]. This expert advantage may reflect the unique cognitive characteristics developed through long-term professional training, including high sensitivity to subtle differences in ball spin direction and trajectory [28,29]. Athletes might develop more efficient perceptual processing strategies through continuous practice and competition experience, enabling them to respond more quickly and accurately [30].

In the analysis of the error rates, the TTAs exhibited higher error rates under incongruent conditions compared to both congruent and category congruent conditions. This suggests that conflict between the priming and target stimuli elevates error rates, even unconsciously [31]. Furthermore, no significant difference was observed in the error rates between the congruent and category congruent conditions, indicating that increased task difficulty did not trigger conflict effects for either the athletes or the non-athletes [32]. Although the group differences in error rates were not significant, increased interference from the priming to the target stimulus notably raised error rates in the incongruent conditions compared to the congruent conditions. This implies that athletes, possibly due to heightened perceptual sensitivity, are more affected by stimulus conflict.

The analysis of the incongruent and category congruent priming effects showed that the athletes had a significantly greater incongruent priming effect at an unconscious level than the non-athletes, which is consistent with prior studies [33]. This indicates that athletes likely possess superior familiarity with the stimulus material and better unconscious processing abilities [33]. Such findings suggest enhanced physiological and cognitive activation in athletes during unconscious perception, potentially due to long-term specialized training [34]. Consequently, it seems plausible that in sports scenarios, athletes rely predominantly on their unconscious perceptual skills to accurately judge the incoming balls’ spin information [33,35]. In contrast, the category congruent priming effect, while greater in the athletes than in the non-athletes, lacked the expected expert advantage, possibly because the increased task difficulty in these conditions impacted the unconscious perception differently. In the incongruent and congruent conditions, differing paddle orientations made the perception less challenging, enhancing the priming effect. However, in the category congruent and congruent conditions, the identical paddle orientation required more complex judgments about the spin and contact angle, increasing the perceptual challenge. Despite this, the athletes still showed a stronger priming effect than the non-athletes, albeit not significantly.

In the ERP analysis, the study revealed the athletes’ temporal characteristics in unconscious information processing. The results showed that in the P2, N1, and N2 components, the TTAs’ ERP amplitudes were significantly lower than those of the non-athletes, deviating from Meng et al.’s (2022) findings [18]. This variation may be due to changes in stimulus material that enhance athletes’ perceptual sensitivity, thus requiring fewer neural resources—a phenomenon aligned with the neural efficiency hypothesis [4]. Individuals with heightened perceptual sensitivity tend to exhibit lower ERP amplitudes and more efficient neural patterns in tasks like serve spin judgment [1,4]. Such efficiency likely derives from specialized cognitive processing skills honed through long-term training, boosting athletes’ performance in processing unconscious information [36]. Athletes’ faster RTs in previous tasks not only confirm their efficient ERP responses but also reflect their overall quicker cognitive speeds, which are attributable to improved information transmission, neural accuracy, and cognitive responsiveness [5]. Consequently, enhanced neural efficiency is a critical factor in athletes’ superior cognitive task performance [36].

The N1 component, which is primarily observed in the occipital region, is essential for selective attention and the initial processing of visual information, making it a key indicator for studying visual perception mechanisms [37]. This study found that both the TTAs and the non-athletes elicited the N1 component during unconscious tasks within the 100–120 ms timeframe. Typically associated with conscious visual processing, the N1 component is also modulated by unconscious stimuli in terms of amplitude and latency, suggesting its role in the deeper processing of visual information [38]. Furthermore, the study reaffirms that the N1 component’s response can be influenced by attention mechanisms, even during unconscious perception, as task-related cues effectively modulate this response [38] [39]. The N1 component is crucial in the early perceptual stages, involving basic feature extraction and preliminary encoding of external stimuli [40]. Its activity during unconscious perception is also shaped by stimulus characteristics, like complexity and relevance, affecting deep visual processing [37]. The TTAs demonstrated greater efficiency with a reduced amplitude of the N1 component, indicating faster and more accurate processing of basic features. Significant priming effects were observed in the amplitude consistency across the O1, Oz, and O2 electrodes, as well as the mean values at the PO3, POz, and PO4 electrodes, indicating a consistent response pattern with the RTs. The athletes displayed both incongruent and category congruent priming effects, unlike the non-athletes, who only showed the incongruent effect. This underscores that as task complexity increases, athletes’ expertise becomes more evident, showcasing their superior ability to process complex visual information [41].

The P2 component, typically observed in the central and parietal regions, is crucial for advanced visual perceptual processing and cognitive evaluation, particularly in assessing the significance and attributes of stimuli [42,43]. In this study, however, both the athletes and non-athletes showed the P2 component in the occipital region within the 150–170 ms timeframe, indicating that the brain continues to analyze information following initial visual processing [43]. Although traditionally linked with the central and parietal regions, the P2 component was found primarily in the occipital region in this study, similar to Yang et al.’s (2017) findings of the P2 component presence in the frontal and occipital regions [44]. This deviation likely results from the different stimuli used; Yang et al.’s (2017) research involved unconscious semantic processing with Chinese characters, whereas our study focused on visual tasks related to the occipital region [44]. In tasks requiring advanced processing, such as interpreting table tennis spin information, particularly under category congruent conditions that enhance visual processing complexity, the P2 component may manifest in the occipital region [42]. Furthermore, the P2 component analysis revealed that the athletes displayed smaller amplitudes in the O1, Oz, and O2 electrodes compared to the control group, suggesting greater neural efficiency in processing complex visual stimuli.

The N2 component, primarily located in the prefrontal cortex, including the central and frontal areas, is also observed in the occipital region under specific conditions [45]. It is associated with deep cognitive control, particularly in inhibiting irrelevant stimuli and resolving task conflicts [46]. Notably, the N2 component’s appearance in the occipital lobe, a central hub for visual processing, signifies activation of cognitive control in visual tasks [46]. This study, exploring unconscious perceptual processing, confirmed the N2’s presence in the occipital lobe within the 190 ms to 210 ms window, aligning with previous findings showing that complex visual information was being processed [47]. Furthermore, the study’s results are consistent with Hughes et al.’s (2009) findings that TTAs show the N2 component under subliminal conditions, highlighting the component’s role in unconscious cognitive control through repetition and inhibition priming tasks [8,46,47]. These tasks illustrate the brain’s ability to enhance or inhibit processing based on prior exposure. The experiment included three conditions: congruent (repetition priming), incongruent (simple inhibition), and category congruent (complex inhibition), revealing significant effects in response consistency and sensitivity of the N2 to inhibition [48]. Notably, under unconscious conditions, the brain effectively manages complex inhibitory tasks, as evidenced by differential N2 responses across conditions, underscoring the brain’s capacity to regulate stimuli responses [7]. The athletes, in particular, demonstrated superior neural efficiency and perceptual sensitivity, as indicated by smaller N2 amplitudes in the table tennis-related tasks, emphasizing their cognitive prowess in processing and inhibiting complex visual information [7].

## 5. Limitations

This investigation serves as a pioneering study into the unconscious perceptual processing of serve spin information among TTAs; yet, it is not devoid of limitations. First, the study is focused exclusively on right-handed participants, which may limit the generalizability of the results to the broader athlete population, including left-handed individuals. Additionally, the study relies heavily on electrophysiological measurements, which, while informative, do not capture the full spectrum of neural activity. The use of only specific electrode sites might also omit relevant data from other areas of the brain involved in neural efficiency. Furthermore, the categorization of participants into only athletes and non-athletes does not account for varying levels of expertise or training within each group, which could influence perceptual processing. Lastly, the cross-sectional design of this study limits our ability to draw conclusions about the causality between long-term training and enhanced neural efficiency. Longitudinal studies are needed to more definitively link training duration and intensity with improvements in unconscious perceptual capabilities.

## 6. Conclusions

This study integrated behavioral and ERP experiments to investigate the characteristics of unconscious information processing by TTAs during serve spin judgment tasks. The results showed that the athletes had shorter RTs and greater priming effects at the behavioral level. Additionally, they displayed higher neural efficiency in the occipital region of the brain, which is attributed to their heightened perceptual sensitivity. This supports the neural efficiency hypothesis, suggesting that long-term professional training has equipped athletes with specialized cognitive processing abilities for serve spin judgment tasks, allowing them to demonstrate superior neural efficiency in processing unconscious information. Extensive repetitive training on serve techniques may have led athletes to develop an intuitive perceptual mechanism at the unconscious level, enabling them to quickly capture essential information. This is especially crucial in sports like table tennis, which demands quick reactive responses and precise judgment. These findings indicate that although conscious performance is vital, the athletes’ advantages at the unconscious level may be a key factor in their success.

## Data Availability

The dataset supporting the conclusions of this article will be made available by the authors on reasonable request. The data are not publicly available due to ongoing analysis and additional research projects that are building upon this dataset.
